# Antibiotic consumption surveillance in rehabilitation facilities – a new task according to § 23 of the German Infection Protection Act. Data from rehabilitation facilities in the Rhine-Main area, 2016–2018

**DOI:** 10.3205/dgkh000365

**Published:** 2020-11-27

**Authors:** Ursel Heudorf, Marlene Berres, Cleo Schmehl, Katrin Steul

**Affiliations:** 1 MDRO Network Rhine-Main, Frankfurt am Main, Germany

**Keywords:** antibiotics, antimicrobial consumption surveillance, rehabilitation facility, neurological early rehabilitation, infection protection law

## Abstract

**Background:** Multidrug-resistant pathogens are a major health problem in many countries. In Germany, in accordance with the German Antibiotic Resistance Strategy (DART), the surveillance of antibiotic consumption in acute care hospitals and rehabilitation facilities was made mandatory by the Infection Protection Act in 2011 and 2017.

Whereas comparable reference data for acute care hospitals are available, such data is lacking for rehabilitation facilities. Therefore, the Rhine-Main network on MDRO (Multi Drug Resistant Organisms) has offered to evaluate the antibiotic consumption of the network's rehabilitation facilities. Antimicrobial consumption (if possible already given as daily defined doses, DDD) and patient days from 2016–2018 were requested.

**Materials and methods:** By October 31, 2019, nine clinics, including a facility for early neurological rehabilitation, reported their consumption of antibiotics (mostly already as DDD) and patient days from 2016–2018. The information from the clinics was entered in an Excel table and the DDD calculated if necessary. In order to compare the facilities, the DDD/100 patient days (pd) were calculated for the individual active substances.

**Results:** Antibiotic consumption in general rehabilitation facilities decreased slightly from 4.8 DDD/100 pd to 4.4 DDD/100 pd from 2016 to 2018. In early neurological rehabilitation, antibiotic consumption increased from 10.2 DDD/100 pd to 13.1 DDD/100 pd in the same period. Among the most commonly used antibiotics, cefuroxime came first, followed by ciprofloxacin and amoxicillin in third place.

**Discussion:** To our knowledge, this is the first antimicrobial consumption data from rehabilitation facilities in Germany. Antibiotic consumption in general rehabilitation facilities is less than 10% of the consumption in normal/regular wards of acute care clinics, and the consumption in neurological early rehabilitation was approximately 10% of the consumption in intensive care wards within acute care clinics. Reserve or broad-spectrum antibiotics were rarely or not used at all in the rehabilitation facilities. Despite this overall positive situation, antibiotic stewardship should also be introduced in rehabilitation facilities, possibly with the support of the regional MDRO networks.

## Zusammenfassung

**Hintergrund:** Multiresistente Erreger sind ein großes Problem in vielen Ländern. In Zusammenhang mit der Deutschen Antibiotika-Resistenz-Strategie (DART) wurden Akutkrankenhäuser und Rehabilitationseinrichtungen in den Jahren 2011 bzw. 2017 durch das Infektionsschutzgesetz (IfSG) verpflichtet, ihren Antibiotika-Verbrauch aufzuzeichnen und zu bewerten. Inzwischen liegen Vergleichsdaten für Akutkrankenhäuser in Deutschland vor, wohingegen Vergleichsdaten für Rehabilitationskliniken bislang fehlen.

Vor diesem Hintergrund hat das MRE-Netz Rhein-Main den Rehabilitationseinrichtungen des Netzwerks angeboten, ihren Antibiotika-Verbrauch auszuwerten. Die Einrichtungen sollten ihren Antibiotika-Verbrauch (möglichst in DDD) und die Patiententage für die Jahre 2016 bis 2018 übermitteln.

**Material und Methode:** Bis zum 31.10.2019 nahmen neun Kliniken incl. einer Einrichtung für neurologische Frührehabilitation das Angebot wahr und übermittelten die wirkstoffbezogenen Verbräuche zumeist als defined daily doses (DDD) und die Patiententage aus den Jahren 2016–2018. Die Angaben der Kliniken wurden in eine Excel-Tabelle eingetragen, bei Bedarf die DDD errechnet. Um die Vergleichbarkeit zwischen den Einrichtungen herzustellen, wurden für die einzelnen Wirkstoffe die DDD/100 Patiententage (PT) berechnet.

**Ergebnisse:** Der Antibiotika-Verbrauch in den allgemeinen Rehabilitationseinrichtungen nahm von 2016 bis 2018 von 4,8 DDD/100 PT auf 4,4 DDD/100 PT leicht ab. In der neurologischen Frührehabilitation nahm der Antibiotikaverbrauch im gleichen Zeitraum von 10,2 DDD/100 PT auf 13,1 DDD/100 PT zu. Unter den am häufigsten eingesetzten Antibiotika stand Cefuroxim an erster Stelle, gefolgt von Ciprofloxacin, an dritter Stelle folgt Amoxicillin.

**Diskussion:** Nach unserer Kenntnis sind das die ersten Antibiotikaverbrauchs-Surveillance-Daten aus Rehabilitationseinrichtungen in Deutschland. Der Antibiotikaverbrauch liegt in den Einrichtungen für allgemeine Rehabilitation (AR) bei deutlich weniger als einem Zehntel des Verbrauchs auf Normalstationen von Akutkliniken, der Verbrauch der Einrichtungen der neurologischen Frührehabilitation (FR) betrug ca. ein Zehntel des Verbrauchs auf Intensivstationen von Akutkliniken. Reserve- oder Breitbandantibiotika wurden in den Rehabilitationseinrichtungen selten bis gar nicht eingesetzt. Trotz dieser insgesamt positiven Situation sollten auch in Rehabilitationseinrichtungen Antibiotic Stewardship-Maßnahmen eingeführt werden, ggf. mit Unterstützung der regionalen MRE-Netzwerke.

## Introduction

Multi-drug-resistant pathogens (MDRO) are a major health problem in many countries. The annual reports of the European Center of Disease Control (ECDC) show the development of resistance in pathogens in European countries. Whereas methicillin-resistant *Staphylococcus aureus* (MRSA) strains are declining, an increase in vancomycin-resistant enterococci (VRE) and multidrug-resistant Gram-negative pathogens (MDRGN) is particularly evident [1]. One way to counter increasing resistance development is to use antibiotics with caution by antibiotic stewardship. The annual ECDC reports on the use of antibiotics in outpatient and inpatient medicine in various European countries [2] exhibit very different antibiotic consumption in different countries, both in terms of quantity and quality, i.e., the use of narrow- and broad-spectrum or reserve antibiotics [2].

In Germany, the German Antibiotic Resistance Strategy (DART) [3] was launched in 2008. So far, two comprehensive reports on antibiotic consumption and the resistance situation in human and veterinary medicine have been published [4], [5]. In accordance with DART, the surveillance of antibiotic consumption in acute care hospitals was made mandatory by the Infection Protection Act in 2011 [6]. In 2013, the Robert Koch Institute (RKI) published guidelines on how this data can be recorded and evaluated [7], [8]. Accordingly, the active ingredients of individual antibiotics should be recorded according to the ATC standard (Anatomical Therapeutic Chemical), specified as DDD (defined daily doses) [9] for every 100 patient days (DDD/100 pd) or 100 cases (DDD/100 cases). This means that not only the consumption of different antibiotics can be compared with each other, but also the trend in consumption in different years or cross-sectionally between different clinics.

Since 2017, rehabilitation facilities have also been obliged to monitor and evaluate their antibiotic consumption in accordance with Section 23 (4) of the Infection Protection Act, to draw conclusions from this and inform the staff [6]. 

For acute care hospitals in Germany as well as other countries comparative data on antibiotic consumption are available, sometimes differentiated between normal and intensive care units or separated into special risk wards, such as haemato-oncological units [5]. Comparable experiences or reference data have so far been lacking for rehabilitation facilities. Therefore, the antibiotic consumption data from rehabilitation facilities in the Rhine-Main area from 2016–2018 were collected. They are reported and discussed below.

## Materials and methods

At the beginning of 2019, the MDRO network Rhine-Main offered to evaluate the antibiotic consumption of the regional rehabilitation clinics and provide them with the results compared to the entirety of all rehabilitation clinics which accepted this offer, but also in anonymous individual comparison. The drug-related consumption (if possible already given as DDD) and the patient days from 2016–2018 were requested. Participation was voluntary.

By October 31, 2019, nine rehabilitation clinics, including a facility for early neurological rehabilitation (ENR), had taken advantage of the offer, with data only from 2017 and 2018 being presented by two clinics. The eight institutions of general rehabilitation (GR) focused on the following: cardiology (n=3), geriatrics, neurology, oncology, orthopedics and urology (each n=1).

The information from the clinics was entered into an Excel table and the DDD calculated. According to the RKI recommendation [7], [8], the DDD/100 patient days (pd) were calculated for the individual active ingredients in order to be able to establish comparability between the facilities. The evaluation was carried out separately for the general rehabilitation clinics (GR) and the rehabilitation facility for early neurological rehabilitation (ENR). To compare the annual antimicrobial consumption (DDD/100 pd) of GR with ENR, the nonparametric Mann-Whitney U-test was applied (SPSS Version 24).

## Results

Antibiotic consumption in general rehabilitation facilities (GR) slightly decreased from 4.8 DDD/100 pd to 4.4 DDD/100 pd (mean values) from 2016 to 2018. In the facility for early neurological rehabilitation (ENR), antibiotic consumption was significantly higher (Mann-Whitney U-Test, p 0.000) and increased from 10.2 DDD/ 100 pd to 13.1 DDD/100 pd from 2016 to 2018. The decrease in GR is due in particular to the decrease in cephalosporin consumption from 1.31 DDD/100 pd in 2016 to 0.9 DDD/100 pd in 2018 (particularly due to the decreasing consumption of cefuroxime from 1.17 DDD/100 pd in 2016 to 0.7 DDD/100 pd). The consumption of the other antibiotic groups remained largely stable. In contrast, the increase in antibiotic consumption in the ENR is mostly due to the increasing consumption of cephalosporins (increase from 1.50 DDD/ 100 PT to 2.54 DDD/100 PT, especially the increasing use of cefuroxime from 0.70 DDD/100 pd to 1.94 DDD/ 100 pd) and quinolones (from 2.0 to 3.3 DDD/100 pd) and to a lesser extent also penicillins (increase from 3.43 DDD/100 pd to 4.08 DDD/100 pd) (Table 1 (Tab. 1)).

Table 1 (Tab. 1) also shows the comparison of antibiotic consumption from 2016 to 2018 in the rehabilitation clinics with the data of the normal and intensive care units from Frankfurt clinics in 2017 [10]. Accordingly, the antibiotic consumption in the GR was less than one-tenth of the consumption in normal wards of acute care hospitals. The consumption of the ENR was about one-tenth of the consumption in intensive care wards in acute care hospitals.

Antimicrobial consumption in the individual rehabilitation facilities differed significantly in some cases. Over all years and all GRs, the total consumption ranged from 1.7 DDD/100 pd to 9 DDD/100 pd, the consumption of penicillins from 0.4 DDD/100 pd to 1.5 DDD/100 pd, that of cephalosporins from 0.2 DDD/100 pd to 3.8 DDD/ 100 pd and quinolone consumption from 0.2 DDD/ 100 pd to 1.9 DDD/100 pd. Apparently, some clinics had already started antibiotic stewardship. For example, two GRs had significantly reduced their total consumption and especially their consumption of cephalosporins.

**Figure 1:**
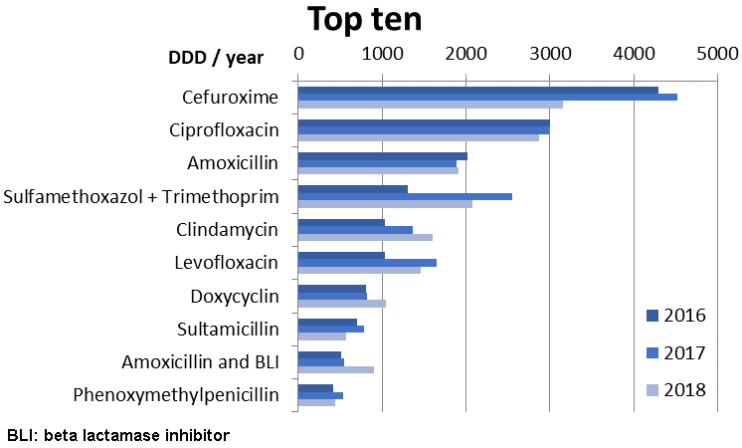
Top 10 antibiotics used in the participating rehabilitation clinics (8 general rehabilitation GR, 1 early neurological rehabilitation ENR) in the years 2016–2018 given in DDD/year (sum)

Figure 1 (Fig. 1) shows the ten antibiotics most commonly used in rehabilitation facilities (GR and ENR combined). Cefuroxime was used most frequently, followed by ciprofloxacin, and amoxicillin. Due to the decrease in the use of cefuroxime in the GR in 2018, the consumption of cefuroxime in 2018 was in the same range as that of ciprofloxacin.

## Discussion

Before discussing the antibiotic consumption data presented here, we would like to point out the study’s limitations. The data originate from a small number of rehabilitation facilities with different rehabilitation indications and only one facility for early neurological rehabilitation (ENR) in a single region in Germany. The comparability of the data to other rehabilitation facilities in other regions in Germany can only be confirmed (or excluded) by further surveys. However, there is no evidence of bias regarding the therapeutic indications of participating rehabilitation facilities.

On the other hand, to our knowledge, this is the first paper on antibiotic consumption data from rehabilitation facilities in Germany – including facilities with various rehabilitation indications. In addition, this is current data, from the past 3 years up to 2018. As long as there are no further publications on antibiotic consumption in rehabilitation facilities available – neither in national nor in international literature –, this data can be used as comparison data for other rehabilitation facilities.

In addition, we suggest that the Robert Koch Institute also enables rehabilitation facilities to participate in its antibiotic consumption monitoring system [11] and offer a module for rehabilitation facilities. For example, the German National Reference Center for Surveillance of Nosocomial Infections has launched special rehabilitation modules for recording hand disinfectant consumption in HAND-KISS [12] and for MRSA surveillance in MRSA-KISS [13], so that rehabilitation facilities can participate, and make open, good comparative data available.

Because hospital antimicrobial consumption data published by the European Centre for Disease Prevention and Control (ECDC) use another denominator (per 1000 inhabitants), our data are not comparable to the European data for methodological reasons [2]. Hence, the data obtained as part of our survey can currently only be compared with data from acute care clinics obtained by the same method, i.e., the antibiotic consumption data from acute care hospitals in Germany 2011 to 2014 published in the GERMAP 2015 report [5] and the more current data from acute care hospitals in the Rhine-Main region (Frankfurt am Main) from 2017 [10]. They show an antibiotic consumption in GR and ENR with approx. 5 DDD/100 pd and 10–13 DDD/100 pd with approx. 10% of the antibiotic consumption in normal and intensive care units, respectively. Penicillins, quinolones and cephalosporins make up the largest part of antibiotics consumed in both GR and ENR facilities. Broad-spectrum or reserve antibiotics such as carbapenems, aminoglycosides or glycopeptides were used rarely or not at all in rehabilitation facilities.

When considering individual active substances, cefuroxime was the most frequently used antibiotic, followed by ciprofloxacin and amoxicillin. This means that one penicillin compound was already the third most often consumed antibiotic and a total of 4 penicillins (2 of which had an enzyme inhibitor) were among the top 10. Cefuroxime is also the most frequently used antibiotic active ingredient in acute hospitals – and in the outpatient sector – in Germany [5], [10], [14]. This active ingredient from the group of second-generation cephalosporins is often used as an IV drug for perioperative prophylaxis. It is also used in oral applications both on an outpatient basis and in clinics. The recommended dose for IV application of cefuroxime is 3x1.5g. Because of the limited oral tolerance (especially diarrhea), recommended oral administration is limited to 2–3x0.5 g. Given the additionally low oral bioavailability of cefuroxime, sufficient and effective active levels in blood plasma cannot be achieved orally. For this reason, the use of cefuroxime (oral) was critically rated as part of the antibiotic stewardship in Frankfurt's acute care hospitals and could thus be reduced by a third from 2012 to 2017 (from 250,398 DDD to 165,160 DDD, i.e. a reduction of 34%) [10].

In the general rehabilitation facilities presented here, cefuroxime consumption decreased by more than a third as well, from 1.17 DDD/100 pd to 0.7 DDD/100 pd from 2016 to 2018, despite the fact that the data was collected retrospectively without previous systematic intervention in the facilities. However, since colleagues from rehabilitation facilities have been attending the Antibiotic Stewardship working group of the MDRO network Rhine-Main [15], [16], [17] where the cefuroxime problem has been discussed in detail, this decrease may well be an effect of a targeted antibiotic stewardship in some rehabilitation clinics.

However, the extensive use of quinolones is of special concern: in 2018 the European Medicines Agency’s human medicines committee (CHMP) recommended restricting the use of fluoroquinolone antibiotics with regard to their possible long-lasting, disabling and potentially permanent side effects involving tendons, muscles, joints and the nervous system. Hence, quinolones should be used with great caution in the elderly, patients with kidney disease, and those who have had an organ transplant, because these patients are at a higher risk of tendon injury. Since the use of a corticosteroid with a fluoroquinolone also increases this risk, combined use of these medicines should be avoided as well [18]. In Germany, a Direct Healthcare Professional Communication, DHPC, was disseminated on April, 8th, 2019 [19]. 

The use of fosfomycin is also currently being discussed very intensively in the working group of the MDRO-network Rhine-Main. Fosfomycin has been used very rarely in rehabilitation clinics. in In the German guideline, fosfomycin-trometamol is recommended for the treatment of uncomplicated urinary tract infections as one of four first-line substances, the others being nitrofurantoin, nitroxolin and pivmecillinam [20]. Since then, the consumption of fosfomycin in outpatient medicine has skyrocketed. The active ingredient is also used in intensive care, mostly in combination with other antibiotics (ß-lactams, carbapenems, aminoglycosides) as a broad-spectrum antibiotic, which acts against MRSA, VRE, 3MRGN and 4MRGN enterobacterales, including some MDR pseudomonas. Fosfomycin also exhibits good activity levels in compartments otherwise difficult to access. Therefore, it was rated as a reserve antibiotic by the World Health Organisation (WHO) and recommended as an antibiotic for difficult-to-treat infections such as bone infections, meningitis or invasive eye infections, but also for the treatment of infections with MDRO [21], [22]. In order to maintain the effectiveness of this substance for the treatment of severe (MDRO) infections, the MDRO network Rhine-Main recommends that fosfomycin should no longer be used orally in clinics and that the other first-line antibiotics for treating uncomplicated community-acquired urinary tract infections should preferably be used, also in rehabilitation facilities 

The comparatively low antibiotic consumption in rehabilitation facilities presented here can also be compared to published point-prevalence studies. As part of the Europe-wide point prevalence study in acute care hospitals in 2016, 25.9% of patients in Germany were treated with antibiotics on the day of the survey (intensive care units 50.2%) [23], [24]. However, point-prevalence data for antimicrobial therapy in rehabilitation facilities in Germany or Europe is lacking. In a current point-prevalence study on multi-drug-resistant pathogens, with 16 rehabilitation facilities in the Rhine-Main region participating in 2019, 3.6% of the 928 patients were treated with antibiotics on the day of the survey, 3.0% of the patients in general rehabilitation facilities, and 21.2% of patients in an early neurological rehabilitation facility [25]. Thus, the results were in the same range as in a survey in 20 rehabilitation facilities in that region in 2014; at that time, 2.9% of the 2440 patients were treated with antibiotics [26]. The use of antibiotics in rehabilitation facilities is somewhat higher than in the nursing homes examined with the same method in 2012 and 2013 in the Rhine-Main area: 1.4%–2.5% of the residents were treated with antibiotics as part of the point-prevalence studies at that time [27], [28]. These data are in line with the European point-prevalence survey of antimicrobial use in long-term care facilities (LTCF): mean prevalence in EU/EEA (European Union/European Economic Area) was 4.9%, but in German LTCFs, antimicrobial use was 1.3% [29].

Nevertheless, further efforts should be made to improve (reduce) the use of antibiotics in rehabilitation facilities. For acute care hospitals, numerous studies have shown that improved, careful antibiotic use not only reduced colonization and infections with multi-resistant pathogens, but also reduced severe Clostridium difficile infections [30], [31], [32]. Given this background, the working group of the medical associations AWMF presented recommendations for antibiotic stewardship in clinics in 2013 and updated them in 2018 [33]. Elements of this guideline should also be implemented in rehabilitation facilities. Here, cooperation between the rehabilitation facilities in a given region may be useful, e.g., in the regional MDRO networks [34] and their antibiotic stewardship activities. An effective collaboration between 27 regional nursing homes for the elderly and external antibiotic stewardship experts was recently reported in the USA: antibiotic consumption (and therefore costs) could be reduced without increasing hospital admissions or readmissions [35].

The MDRO networks in Germany not only advise institutions – including rehabilitation facilities – on questions about multi-resistant pathogens. Most MDRO networks have also started projects on the responsible use of antibiotics and offer information flyers on this. The MDRO network Rhine-Main for example, offers flyers on the responsible use of antibiotics for respiratory infections, urinary tract infections, and ear infections. For instance, with these flyers, patients or relatives can be informed that 80% of respiratory infections are caused by viruses and antibiotics do not help, or that 70% of uncomplicated urinary tract infections heal even without antibiotics. The flyers also provide information on suitable “home remedies”. They were created in cooperation with the relevant professional medical associations, based on current guidelines, and are available free of charge on the homepage or can be ordered from the network [36].

## Conclusion and key messages

In Germany, rehabilitation facilities have been obliged to monitor their antibiotic consumption since 2017. In this paper, antibiotic consumption data from the years 2016–2018 from rehabilitation facilities in Germany are presented for the first time.

Antibiotic consumption in the rehabilitation facilities was generally less than 5 DDD/100 pd, but in a facility for early neurological rehabilitation between 10 and 13 DDD/100 pd. This is about 10% of the consumption in acute care hospitals (normal wards approx. 56 DDD/ 100 pd, intensive care units approx. 120 DDD/100 pd [data from Frankfurt am Main]). Broad-spectrum or reserve antibiotics are prescribed very rarely or not at all.

Even if antibiotic consumption in the rehabilitation facilities was low compared to that in acute care clinics, antibiotic stewardship should also aim to reduce and improve the use of antibiotics in rehabilitation facilities. This article offers suggestions to this end, e.g., cooperation with the regional MDRO networks.

## Notes

### Competing interests

The authors declare that they have no competing interests.

## Figures and Tables

**Table 1:**
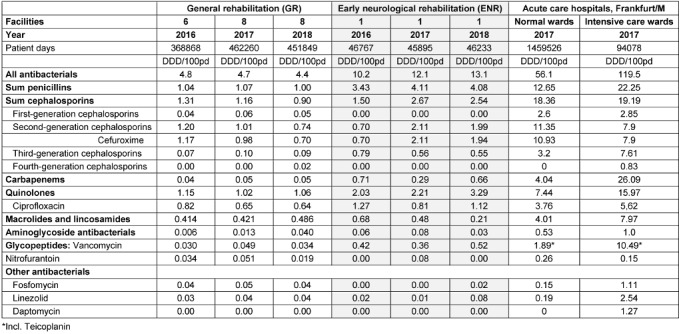
Antibiotic consumption data (DDD/100 patient days) for 2016–2018 in 8 general rehabilitation facilities and one facility for early neurological rehabilitation, compared to 2017 antibiotic consumption data from the normal and intensive care units of acute care hospitals in Frankfurt am Main, Germany [10].
